# CircRNA RSF1 regulated ox-LDL induced vascular endothelial cells proliferation, apoptosis and inflammation through modulating miR-135b-5p/HDAC1 axis in atherosclerosis

**DOI:** 10.1186/s40659-021-00335-5

**Published:** 2021-03-23

**Authors:** Xiaohao Zhang, Junying Lu, Qinghua Zhang, Qiang Luo, Bin Liu

**Affiliations:** 1 Department of Cardiology, The Second Hospital of Jilin University, No.218, Ziqiang Street, Nanguan District, Changchun, 130041 Jilin China; 2 Department of Intensive Care Unit, The First Hospital of Jilin University, Changchun, Jilin China; 3 Respiratory and Critical Illness Department, The Second Hospital of Jilin University, Changchun, Jilin China

**Keywords:** CircRNA RSF1, MiR-135b-5p, HDAC1, Cell progression, Inflammation, Atherosclerosis

## Abstract

**Background:**

Atherosclerosis (AS) is the most common type in cardiovascular disease. Due to its complex pathogenesis, the exact etiology of AS is unclear. circRNA has been shown to play an essential role in most diseases. However, the underlying mechanism of circRNA in AS has been not understood clearly.

**Methods:**

Quantitative Real-Time PCR assay was used to detect the expression of circRSF1, miR-135b-5p and histone deacetylase 1 (HDAC1). Western blot was applied to the measure of protein expression of HDAC1, B-cell lymphoma-2 (Bcl-2), BCL2-associated X (Bax), cleaved-caspase-3, vascular cell adhesion molecule 1 (VCAM1), intercellular cell adhesion molecule-1 (ICAM1) and E-selectin. MTT assay and flow cytometry were used to detect cell proliferation and apoptosis, respectively. Dual luciferase reporter assay and RIP assay was used to determine the relationship among circRSF1, miR-135b-5p and HDAC1. Besides, an ELISA assay was performed to measure the levels of IL-1β, IL-6, TNF-α and IL-8.

**Results:**

In this study, ox-LDL inhibited circRSF1 and HDAC1 expression while upregulated miR-135b-5p expression in Human umbilical vein endothelial cells (HUVECs). Importantly, ox-LDL could inhibit HUVECs growth. Moreover, promotion of circRSF1 or inhibition of miR-135b-5p induced cell proliferation while inhibited apoptosis and inflammation of ox-LDL-treated HUVECs, which was reversed by upregulating miR-135b-5p or downregulating HDCA1 in ox-LDL-treated HUVECs. More than that, we verified that circRSF1 directly targeted miR-135b-5p and HDAC1 was a target mRNA of miR-135b-5p in HUVECs.

**Conclusion:**

CircRSF1 regulated ox-LDL-induced vascular endothelial cell proliferation, apoptosis and inflammation through modulating miR-135b-5p/HDAC1 axis in AS, providing new perspectives and methods for the treatment and diagnosis of AS.

## Introduction

Atherosclerosis (AS) is one of the most common vascular diseases and is the main cause of coronary heart disease, cerebral infarction, and peripheral vascular disease [1–3]. The pathogenesis of AS is not clear and has not yet been fully elucidated. In addition, AS has a high disability and mortality rate, thus prevention and early diagnosis are critical. Elevated low-density lipoprotein (LDL) is an important risk factor for AS [4, 5]. In addition, after oxidative modification, oxidative (ox)-LDL promoted AS more severely. Previous studies had shown that ox-LDL treatment inhibited the proliferation of vascular endothelial cells, promoted the apoptosis of endothelial cells, and induced the inflammation and oxidative stress of endothelial cells [4, 6, 7].

Circular RNAs (circRNAs) are a type of circular non-coding RNA formed by the back-splicing, and are the resistance of RNA exonuclease as well as maintain stability in eukaryotic cells [8, 9]. At present, accumulating evidence showed that circRNA played an important regulatory role in the occurrence and development of diseases [10–14]. CircRNA regulated the expression of target genes through binding to miRNA and played a key role in the entire regulatory network [15–17]. For example, circRNA_010567 was highly expressed in myocardial fibrosis and contributed to myocardial fibrosis via targeting miR-141/ TGF-β1 axis [18]. Deng et al. reported that hsa_circ_0009910 induced carcinogenesis through targeting miR-449a to regulate IL6R in osteosarcoma [19]. Moreover, hsa_circ_0000345 (circRSF1) was a fragment localized to the remodeling and spacing factor 1 (RSF1) gene, which was down-regulated in HUVECs treated with ox-LDL (100 mg/L) [20]. However, the specific regulatory mechanism and function of circRSF1 have not been fully understood in AS.

MicroRNAs (miRNAs) are short, single-strand non-coding RNAs containing ~ 22nt that regulate mRNA expression at post-transcriptional level by binding 3′UTR of target mRNA [21]. Various studies have shown that miRNAs were involved in cell progression of cancers, including AS [22–24]. Importantly, miR-135b-5p was an important regulation factor in cancers and was related to cell proliferation, apoptosis, migration and invasion [25, 26]. More than that, miR-135b-5p also related to cell proliferation and migration through targeting myocyte enhancer factor 2C (MEF2C)[27]. miR-135a also inhibited oxidative stress and vascular inflammatory in OA [28]. Since miRNA could target multiple mRNAs and then function, the regulatory network of miR-135b-5p still needs to be further explored.

Histone deacetylase 1 (HDAC1) is a protein-coding gene located on chromosome 1 in the human genome [29]. Many evidence showed that HDAC1 was associated with affected disease progression, including cell proliferation and apoptosis [30, 31]. For example, in HDAC1/2 double mutant mice, activation of the Wnt/β-catenin pathway accelerates the inhibition of oligodendrocyte differentiation [30]. Besides, knockdown of HDAC1,2 could promote cell apoptosis in chronic myeloid leukemia cells [32]. However, the regulatory mechanism of HDAC1 in AS has not been clear.

In this study, we found that circRSF1 could sponge miR-135b-5p to target HDAC1 in AS. Through a series of cell physiology and biochemistry experiments, we demonstrated that circRSF1/miR-135b-5p/HDAC1 axis played an essential role in AS, providing a new target and perspective for AS treatment.

## Materials and methods

### Cell culture and treatment

Human umbilical vein endothelial cells (HUVECs) were purchased from the American Type Culture Collection (ATCC, Manassas, VA, USA) and cultured in Dulbecco’s modified Eagle medium (DMEM, Thermo Fisher Scientific, Inc. USA) with 10% FBS and penicillin/streptomycin at 37 °C atmospheres with 5% CO_2._ To establish the AS model, HUVECs were treated with 100 μg/mL ox-LDL(Sigma-Aldrich, St. Louis, MO) for 48 h.

### The detail of circular RNA RSF1 (circRSF1)

The circRNA ID of circRSF1 was hsa_circ_0000345, which was derived from RSF1 gene. The position of circRSF1: Chr11:77409531–77413540.

### Cell transfection

CircRSF1 overexpression (circRSF1), HDAC1 inhibitor (si-HDAC1), miR-135b-5p mimics (miR-135b-5p), miR-135b-5p inhibitor (anti-miR-135-5p) and their negative control (NC; vector, si-NC, miR-NC and anti-miR-NC) were purchased from GenePharma (Shanghai, China). The vectors and oligonucleotides (50 nM) were transfected into ox-LDL induced HUVECs using the Lipofectamine 2000 reagent (Invitrogen, Carlsbad, CA, USA). All transfections experiments were performed in triplicates.

### The sequences of siRNA, miRNA and negative controls

Overexpression of circRSF1 was 1200 bp upstream and 500 bp downstream sequence was added to nonlinear splice sites of circRSF1, and the correct fragment was amplified and inserted into the pcDNA3.1 vector. MiR-135b-5p inhibitor: 5′- UCACAUAGGAAUGAAAAGCCAUA -3′;

si-HDAC1: sense: 5′-GCCGGUCAUGUCCAAAGUATT-3′, antisense: 5′-UACUUUGGACAUGACCGGCTT-3’.

miR-135b-5p: 5′-UAU GGC UUU UUA UUC CUG UGU GA-3’

miR-NC: 5′- CAGUACUUUUGUGUAGUACAA -3′;

Si-NC: sense: 5′-GAA UUA AUU AAA GAU GGC CCG UUG UAC U-3′; antisense: 5′-UCA UCG AAG UUA UAG GGA UAC AUU ACG UGA UC-3’

anti-miR-NC: 5′-UUU GUA CUA CAC AAA AGU ACU G-3’

### Quantitative real-time PCR

Total RNA was isolated from cells using TRIzol reagent (Invitrogen). The TaqMan® MicroRNA Real Time-PCR Assay reagents (Applied Biosystems; Foster City, CA, USA) and the stem-loop primer SYBR Green quantitive real time-PCR (RiboBio, Guangzhou, China) were used to detect miR-135b-5p expression. The High Capacity cDNA Reverse Transcription Kit (Applied Biosystems) and the SYBR Green PCR Kit (Takara, Otsu, Japan) were performed to measure circRSF1, RSF1 and HDAC1 expression. CircRSF1, RSF1 and HDAC1 expression were normalized to GAPDH. MiR-135b-5p was normalized to U6. QRT-PCR was conducted: Hold 50 °C for 2 min, 95.0 °C for 20 s, and 40 circles of 95.0 °C for 15 s and 60 °C for 20 s. The 2^−ΔΔCt^ method was used to quantify miR-135b-5p, circRSF1, RSF1 and HDAC1 expression. All experiments were performed in triplicates.

circRSF1 Forward: 5′-AAAGTGAAGGGTCTGGCAGT-3’

circRSF1 Reverse: 5′-ACTGGCAGTTTCACAAGTTCC-3’

RSF1 Forward: 5′-GATACTATGCGTCTCCAGCCAA-3’

RSF1 Reverse: 5′-CAACTCGTTTCGATTTCTGACAA-3’

miR-135b-5p Forward: 5′-GGGCGTATGGCTTTTCA-3’

miR-135b-5p Reverse: 5′-CAGTGCAGGGTCCGAGGTA-3’

HDAC1 Forward: 5′-CTACTACGACGGGGATGTTGG-3’

HDAC1 Reverse: 5′-GAGTCATGCGGATTCGGTGAG-3’

U6 Forward: 5′- CGCTTCGGCAGCACATATAC-3’

U6 Reverse: 5′- TTCACGAATTTGCGTGTCAT-3’

GAPDH Forward 5′-TCAAGGCTGAGAACGGGAAG-3’

GAPDH Reverse 5′-TGGACTCCACGACGTACTCA-3’

### Actinomycin D and RNase R treatment

Actinomycin D (2 mg/mL, Sigma Aldrich, St. Louis, MO, USA) was added into the DMEM medium to detect the stability of circRSF1 and its linear isoform.

Total RNA (1 μg) was incubated with RNase R (1U, Epicentre, Madison, Wisconsin, USA) at 37 °C for 30 min. Then, the expression of circRSF1 and RSF1 was detected with qRT-PCR. All experiments were performed in triplicates.

### RNA pull-down

Briefly, miRNA related fragment (miR-135b-5p-WT, miR-135b-5p-MUT) were incubated with biotin (Bio)-labeled oligonucleotide probes of miR-135b-5p (Bio-5′-TTAAACCAACATCTTTTCTGACACAGAGACGGCG-3′, RiboBio, Guangzhou, China) and the RIP lysates against circRSF1 for 2 h at 25 °C. circRSF1 and miR-135b-5p complexes were collected with Streptavidin-coupled Dynabeads (Invitrogen) and then incubated with binding wash buffer (20 mM Tris(pH7.5) 、150 mM NaCl、1%Triton X-100、2 mM DTT、1 mM EDTA) containing proteinase K for 1 h at 25 °C. The circRSF1 was determined using qRT-PCR analysis. Each experiment was performed in triplicate.

### Western blot

Transfected cells were lysed in RIPA buffer (Beyotime, Haimen, China) to extract total protein. The protein concentration was measured using the BCA™ Protein Assay Kit (Pierce, Appleton, WI, USA). An equal amount of proteins (50 µg) were added onto the SDS-PAGE to separate the target protein. And then the target protein was transferred onto the polyvinylidene difluoride (PVDF) membrane (Millipore, Billerica, MA, USA). Next, the membrane was incubated with primary antibodies against B-cell lymphoma-2 (Bcl-2), BCL2-associated X (Bax), cleaved-caspase-3, vascular cell adhesion molecule 1 (VCAM1), intercellular cell adhesion molecule-1 (ICAM1), E-selectin, HDAC1 and GAPDH (1:1,000, ProteinTech, Chicago, IL, USA) at 4℃ overnight. After washed in TBST, the membrane was incubated with horseradish peroxidase (HRP)-conjugated goat anti-rabbit secondary antibody (Santa Cruz Biotechnology, Santa Cruz, CA, USA). The blot intensities were detected and analyzed using an ECL Plus Western Blotting Substrate (Thermo Fisher Scientific, Inc., Waltham, MA, USA) and Image-Pro Plus software.

### MTT assay

The proliferation viability of transfected cells was measured using 3-(4,5-dimethyl-2-thiazolyl)-2,5-diphenyl-2-H-tetrazolium bromide (MTT; Sigma-Aldrich) assay. The cells were added to 96 well plates. 20 µL MTT solution (5 mg/mL) was added to the plate and incubated at 37 ° for 4 h, then 150 µL of DMSO was added to each well. Put the plate into the incubator at 37 ° for 5 min. The absorbance at 490 nm was recorded to calculate cell proliferation rate using a microplate reader (BioTek, Winooski, VT, USA).

### Cell apoptosis

Cell apoptosis was analyzed using a PI/Annexin V-FITC Apoptosis Detection Kit (BD Biosciences, San Jose, CA) by Gallios Flow Cytometer (Beckman Coulter, Chaska, MN, USA) according to the manufacturer’s instructions. In brief, the cells were digested with trypsin. After washed in PBS, cells were double-stained with Annexin V/FITC and Propidium Iodide (PI) at 37 °C for 15 min in darkness.

### ELISA

Enzyme-linked immunosorbent assay (ELISA, Beyotime Biotechnology, Shanghai, China) was performed to assess levels of interleukin (IL)-1β, IL-6, IL-8 and tumor necrosis factor (TNF)-α according to the manufacturer’s instructions.

### Dual luciferase reporter assay

The web of circbank was used to predict the target miRNA of circRSF1 and the starBase v2.0 was used to predict the target mRNA of miR-135b-5p in HUVECs.The wild type sequences and mutated type sequences (mutate binding sites of miR-135b-5p) of circRSF1 and HDAC1 were inserted into the pGL3 promoter vector (Promega, Madison, WI, USA) to construct the vector of circRSF1 wild-type (WT)/mutated(MUT) and HDAC1 3′-UTR-WT/MUT. These vectors were co-transfected with miR-135b-5p or miR-NC into ox-LDL induced HUVECs using the Lipofectamine 2000 reagent (Invitrogen). After transfection for 48 h, the luciferase activities were detected using Dual-Luciferase Reporter Assay System (Promega). *Renilla* luciferase activities are normalized to firefly luciferase activities.

### RNA immunoprecipitation (RIP assay)

RIP was performed using EZ-Magna RIP Kit (Millipore, Billerica, MA, USA) according to the protocols of manufacturers. The cells transfected with miR-135b-5p mimics and circRSF1 were lysed in RIP lysis buffer with proteinase and RNase inhibitors. The lysates were coincubated with 100µL RIP buffer with A/G magnetic beads conjugated with anti-Ago2 or anti-IgG (Cell Signaling Technology, Danvers, MA, USA) and then the protein was digested using Proteinase K buffer. The enrichment of miR-135b-5p and circRSF1 in the immunoprecipitated RNA was measured using qRT-PCR.

### Statistical analysis

Statistical analysis was performed using Prism 7.0 software (GraphPad Software Inc., La Jolla, CA, USA) and the data were presented as mean ± standard deviation (SD). Chi-squared test, Student’s t-test and Wilcoxon signed-rank test were performed for comparison, as appropriate. One-way ANOVA was used to analyze statistical significance of three and more groups. P values at 0.05 or smaller were considered statistically significant. Each experiment was performed in triplicate.

## Results

### Ox-LDL inhibited circRSF1 expression in HUVECs

**Fig. 1:**
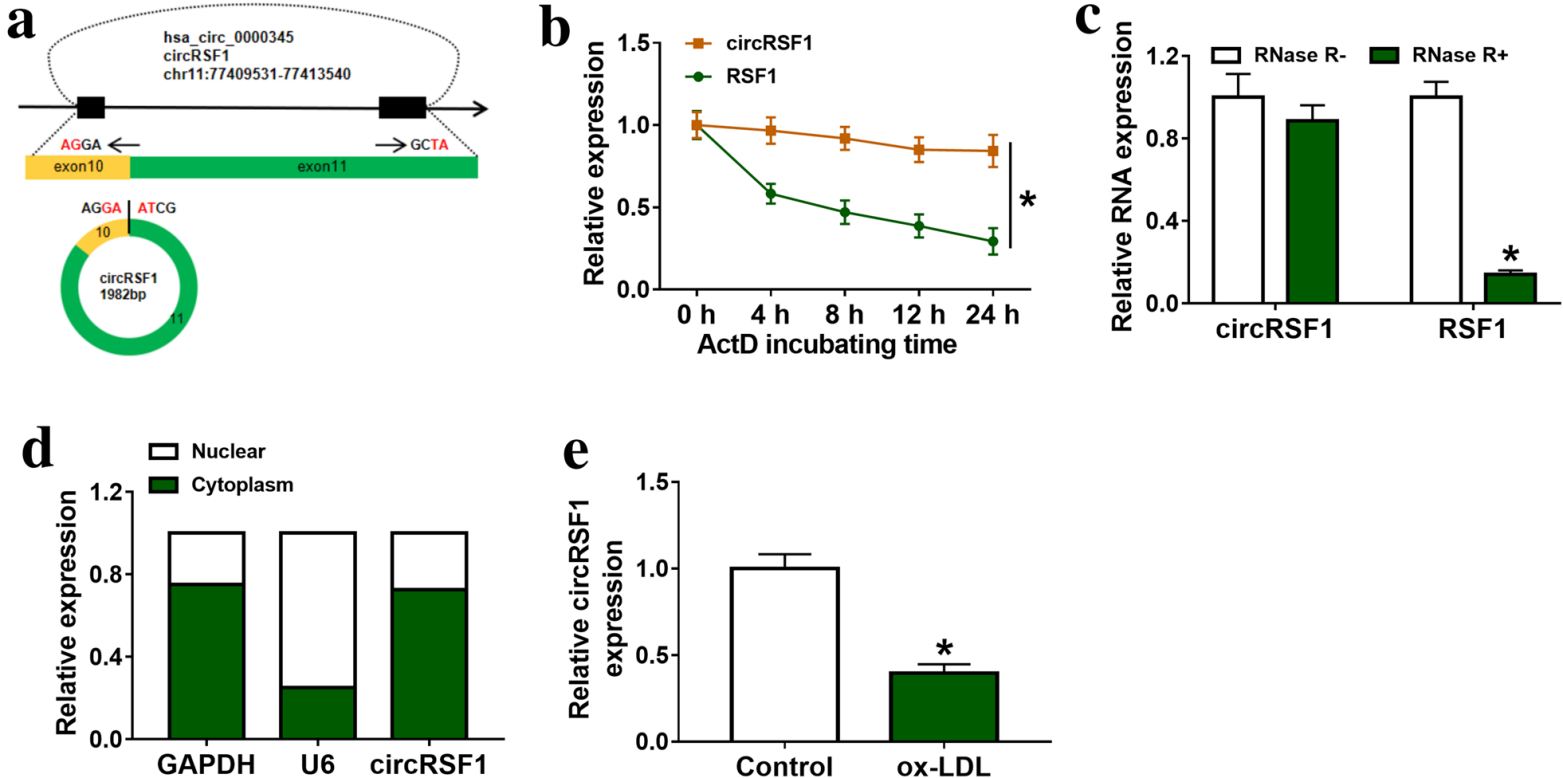
Oxidative (ox)-LDL(Ox-LDL) inhibited circRSF1 expression in HUVECs. **a** The graph of genomic loci of RSF1 gene and circRSF1. **b** The effect of actinomycin D (ActD) on cells was detected by qRT-PCR, and the abundances of circRSF1 and RSF1 mRNA were detected at specified time points. **c** RNase R digestion test was used to test the stability of circRSF1. After RNase R treatment, the expression of circRSF1 was more stable than RSF1 mRNA. **d** The expression of circRSF1 in nuclear and cytoplasmic of HUVECs **e** the expression of circRSF1 was detected in Control and ox-LDL of HUVECs. Each experiment was performed in triplicate *p < 0.05

CircRSF1 is derived from the RSF1 gene exon 10–11, and its mature sequence length is 1982 bp (Fig. 1a). HUVECs were treated with actinomycin and the expressions of circRSF1 and RSF1 were determined at 0, 4, 8, 12 and 24 h by qRT-PCR. The results showed that circRSF1 was more stable than linear RSF1 (Fig. 1b). After treatment with RNase R in HUVECs, we determined that circRSF1 was resistant to RNase R (Fig. 1c). Furthermore, nuclear and cytoplasmic separation demonstrated that circRSF1 was mainly located in cytoplasm of HUVECs (Fig. 1d). As shown in Fig. 1e, compared with control group, the expression of circRSF1 was significantly lower in ox-LDL groups (Fig. 1e). Therefore, ox-LDL suppressed circRSF1 expression in HUVECs.

### Induction of circRSF1 promoted cell proliferation and inhibited apoptosis and inflammation of ox-LDL-treated HUVECs

**Fig. 2:**
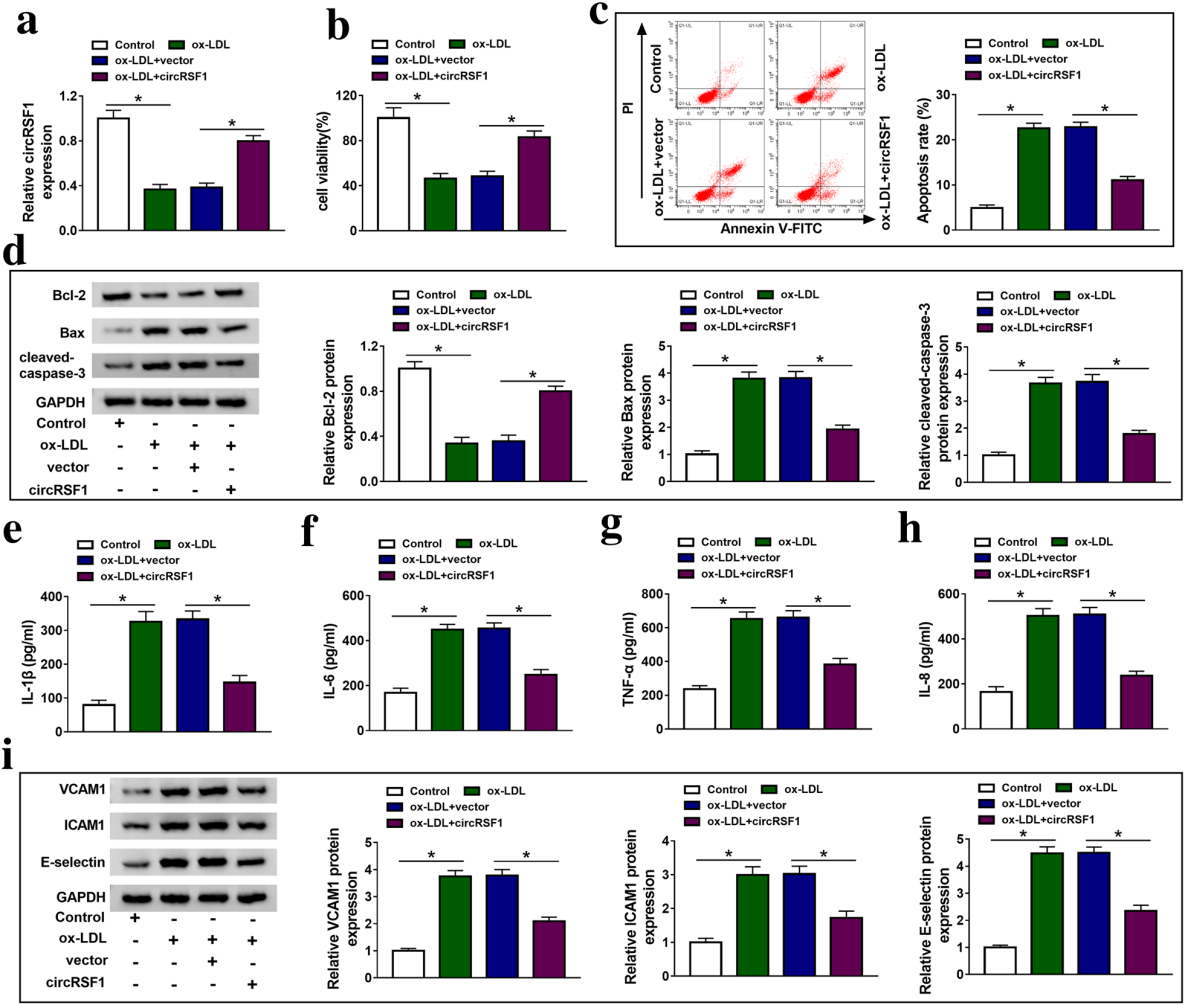
Induction of circRSF1 promoted cell proliferation, and inhibited apoptosis and inflammatory of ox-LDL-treated HUVECs. **a** The expression of circRSF1 was measured in control, ox-LDL, ox-LDL + vector and ox-LDL + circRSF1 groups using qRT-PCR. **b** MTT assay was applied to measure cell proliferation in control, ox-LDL, ox-LDL + vector and ox-LDL + circRSF1 groups. **c** Flow cytomety was used to detect cell apoptosis in control, ox-LDL, ox-LDL + vector and ox-LDL + circRSF1 groups. **d** Western blot assay detected the protein expression of Bcl-2, Bax and cleaved-caspase-3 in control, ox-LDL, ox-LDL + vector and ox-LDL + circRSF1 groups. **e**–**h** ELISA was used to assess the level of IL-1β, IL-6, TNF-α and IL-8 in control, ox-LDL, ox-LDL + vector and ox-LDL + circRSF1 groups. **i** The protein expression of VCAM1, ICAM1 and E-selectin was detected in control, ox-LDL, ox-LDL + vector and ox-LDL + circRSF1 groups. ‘ + ’ was added, ‘−’ was blank *p < 0.05

To explore the function of circRSF1 in ox-LDL-treated HUVECs, circRSF1 and vector were transfected into ox-LDL-treated HUVECs and the results showed that circRSF1 transfection induced the expression of circRSF1 in ox-LDL-treated HUVECs (Fig. 2a). MTT assay demonstrated that ox-LDL inhibited cell viability in HUVECs and overexpression of circRSF1 could improve cell viability in ox-LDL-treated HUVECs (Fig. 2b). Moreover, cell apoptosis was induced by ox-LDL treatment, which was inhibited by circRSF1 transfection in HUVECs (Fig. 2c). In addition, ox-LDL inhibited Bcl-2 protein expression and induced Bax and cleaved-caspase-3 protein expression in HUVECs. Overexpression of circRSF1 significantly promoted Bcl-2 protein expression, and decreased Bax and cleaved-caspase-3 protein expression in ox-LDL-treated HUVECs (Fig. 2d). ELISA assay was used to detect the level of inflammatory factors, including IL-1β, IL-6, TNF-α and IL-8. As shown in Fig. 2e–h, ox-LDL treatment enhanced IL-1β, IL-6, TNF-α and IL-8 level in HUVECs, while circRSF1 transfection sharply reduced the level of IL-1β, IL-6, TNF-α and IL-8 in ox-LDL-treated HUVECs. VCAM1, ICAM1 and E-selectin are adhesion molecules that can reflect cellular inflammation. The results of western blot showed that ox-LDL induced VCAM1, ICAM1 and E-selectin protein expression in HUVECs, but overexpression of circRSF1 could repress the protein of VCAM1, ICAM1 and E-selectin in ox-LDL-treated HUVECs (Fig. 2i). These data implied that the promotion of cirRSF1 could weaken the inhibitory effect of ox-LDL treatment on HUVECs. Therefore, circRSF1 overexpression promoted cell proliferation, inhibited apoptosis and inflammation of ox-LDL-treated HUVECs, suggesting that circRSF1 was associated with AS and played important roles in AS.

### CircRSF1 directly targeted miR-135b-5p in HUVECs

**Fig. 3:**
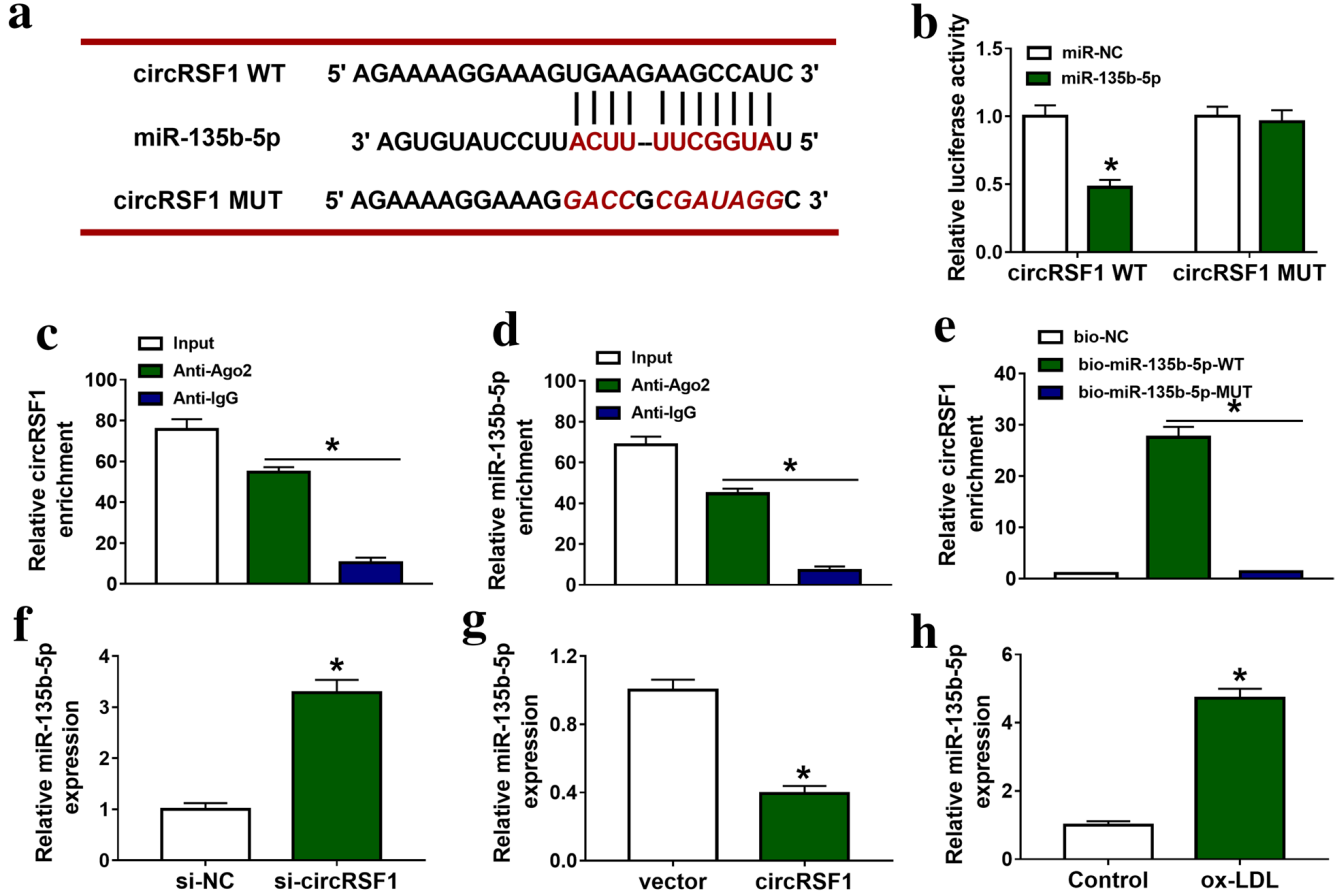
CircRSF1 directly targeted miR-135b-5p in HUVECs. **a** The predicted binding sites of miR-135b-5p to the circRSF1 sequences using circbank. **b**–**d** Dual luciferase reporter assay (**b**), RIP assay (**c**, **d**) and pull down (**e**) determined that miR-135b-5p was a target miRNA of circRSF1 in HUVECs. **f** qRT-PCR was used to detect the expression of miR-135b-5p in si-NC and si-circRSF1 groups, vector and circRSF1 groups (**g**), control and ox-LDL groups **h** in HUVECs. Each experiment was performed in triplicate *p < 0.05

To further realize the regulatory network of circRSF1 in AS, we found that circRSF1 had reverse complementary sites to miR-135b-5p in HUVECs (Fig. 3a). Dual luciferase reporter assay showed that when the miR-135b-5p bound to circRSF1 WT, luciferase activity was notably decreased in HUVECs (Fig. 3b). More than that, RIP assay determined that circRSF1 and miR-135b-5p could bind to Ago2 protein (Fig. 3c, d). The results of pull down showed that the mutated miR-135-5p failed to trigger circRSF1 enrichment (Fig. 3e). As shown in Fig. 3f, g, silencing or promoting circRSF1 expression could improve or inhibit the expression of miR-135b-5p in HUVECs. qRT-PCR analysis showed that ox-LDL could promote miR-135b-5p expression in HUVECs (Fig. 3h). Therefore, all data determined that miR-135b-5p was a target miRNA of circRSF1.

### The effects of high circRSF1 expression on cell proliferation, apoptosis and inflammatory of ox-LDL-treated HUVECs was reversed by miR-135b-5p overexpression

**Fig. 4:**
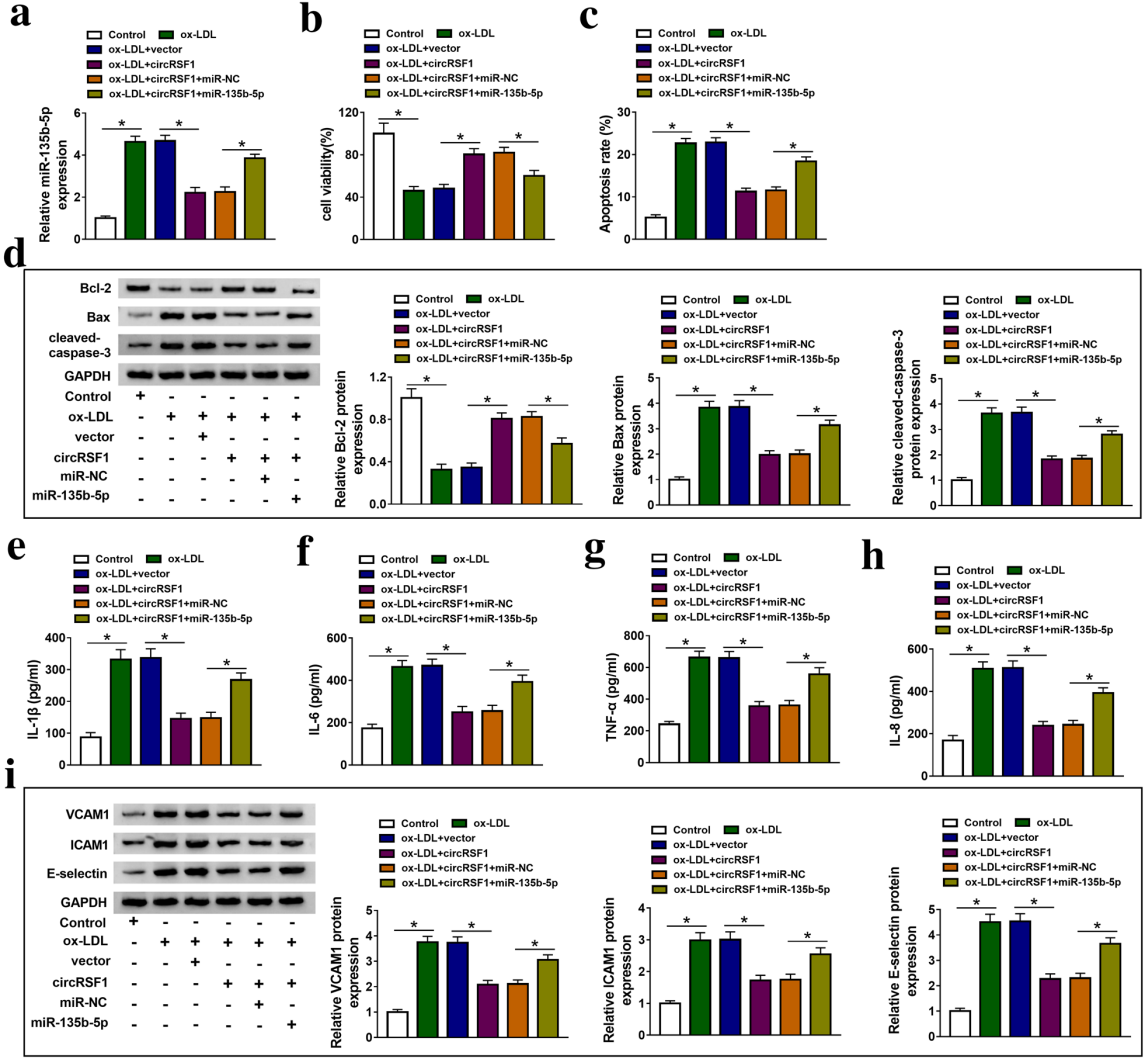
The effects of high circRSF1 expression on cell proliferation, apoptosis and inflammatory of ox-LDL-treated HUVECs was reversed by miR-135b-5p overexpression. **a** The expression of miR-135b-5p was measured in control, ox-LDL, ox-LDL + vector, ox-LDL + circRSF1, ox-LDL + circRSF1 + miR-NC and ox-LDL + circRSF1 + miR-135b-5p groups using qRT-PCR, the measurements of the following indicators are grouped in this experiment. **b** MTT assay was applied to measure cell proliferation. **c** Flow cytometry was used to detect cell apoptosis. **d** Western blot assay detected the protein expression of Bcl-2, Bax and cleaved-caspase-3. **e**–**h** ELISA was used to assess the level of IL-1β, IL-6, TNF-α and IL-8 in control, ox-LDL, ox-LDL + vector, ox-LDL + circRSF1, ox-LDL + circRSF1 + miR-NC and ox-LDL + circRSF1 + miR-135b-5p groups. **i** Western blot assay detected the protein expression of VCAM1, ICAM1 and E-selectin. ‘ + ’ was added, ‘−’ was blank. Each experiment was performed in triplicate *p < 0.05

In order to further understand the regulation mechanism of circRSF1 and miR-135b-5p on ox-LDL-treated HUVECs, we conducted rescue experiments. qRT-PCR results showed that circRSF1 transfection inhibited the expression of miR-135b-5p in ox-LDL-treated HUVECs, while overexpression of miR-135b-5p significantly reduced this effect (Fig. 4a). Consistent with the foregoing results, promotion of circRSF1 enhanced cell proliferation, and inhibited apoptosis and inflammation of ox-LDL-treated HUVECs (Fig. 4b–i). MTT assay determined that inducing miR-135b-5p expression could inhibit cell proliferation in ox-LDL-treated HUVECs promoted by circRSF1 transfection (Fig. 4b). Similarly, cell apoptosis and the level of IL-1β, IL-6, TNF-α and IL-8 were remarkably decreased by upregulating circRSF1 expression, which were rescued by miR-135b-5p transfection (Fig. 4c, e–h). Furthermore, the inhibitory or stimulative effect of high circRSF1 expression on Bax, cleaved-caspase-3, VCAM1, ICAM1 and E-selectin or Bcl-2 protein expression was significantly alleviated by the promotion of miR-135b-5p in ox-LDL-treated HUVECs (Fig. 4d and i). Therefore, circRSF1 regulated cell proliferation, apoptosis and inflammation through targeting miR-135b-5p in ox-LDL-treated HUVECs.

### HDAC1 was a target mRNA of miR-135b-5p in HUVECs

**Fig. 5:**
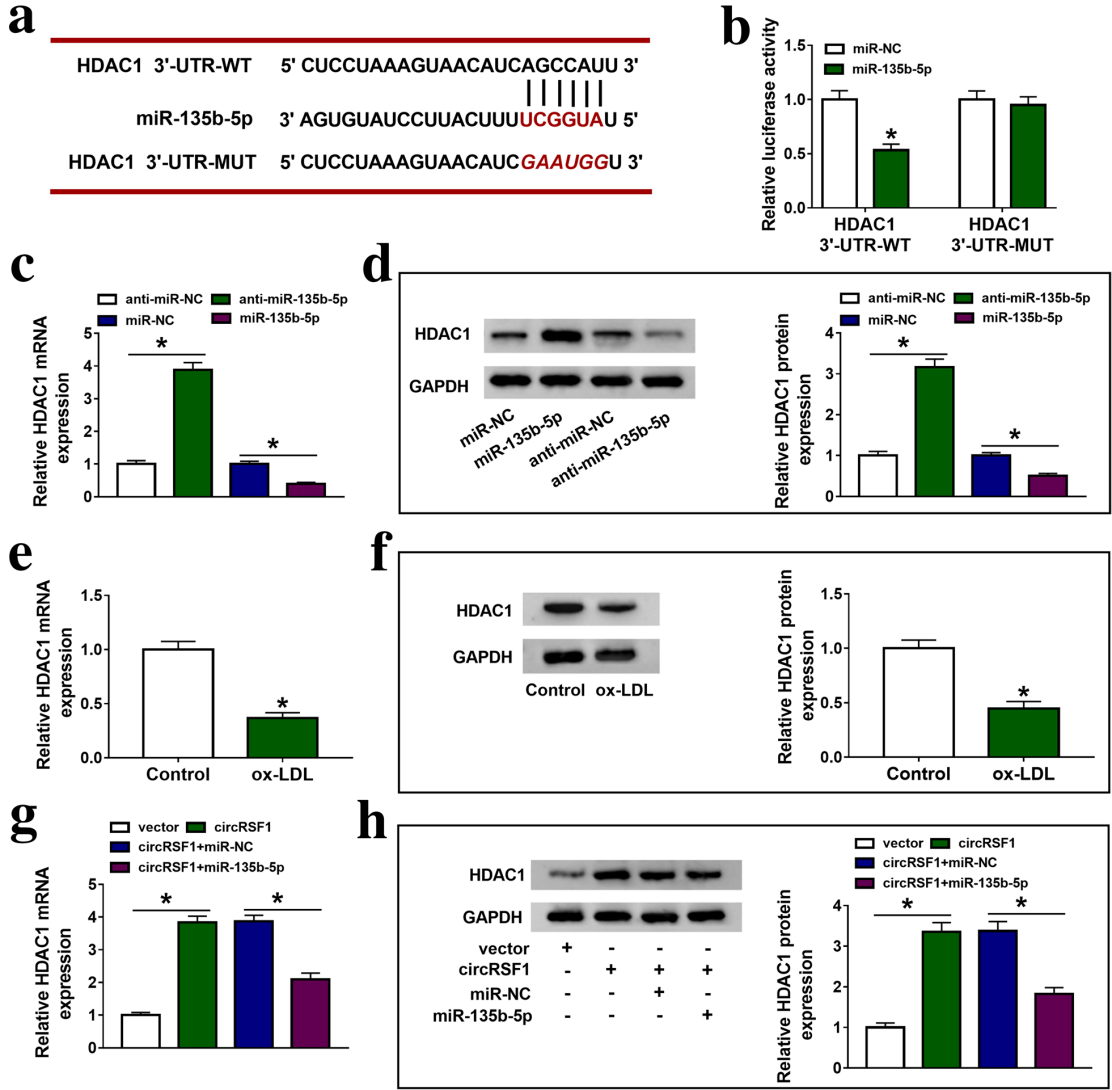
HDAC1 was a target mRNA of miR-135b-5p in HUVECs. **a** The predicted binding sites of miR-135b-5p to the HDAC1 sequences using circbank. **b** Luciferase reporter assay determined that miR-135b-5p was a target miRNA of circRSF1 in HUVECs. **c**, **d** qRT-PCR and western blot were used to detect the mRNA and protein expression of HDAC1 in miR-NC, miR-135b-5p, anti-miR-NC and anti-miR-135b-5p groups in HUVECs. **e**, **f** qRT-PCR and western blot were used to detect HDAC1 mRNA and protein expression in control and ox-LDL groups in HUVECs. **g**, **h** qRT-PCR and western blot were used to detect HDAC1 mRNA and protein expression in vector, circRSF1, circRSF1 + miR-NC, circRSF1 + miR-135b-5p groups in HUVECs. Each experiment was performed in triplicate *p < 0.05

Next, we found that HDAC1, which contained binding sites with miR-135b-5p, was a potential target mRNA of miR-135b-5p. (Fig. 5a). Dual luciferase reporter assay showed that miR-135b-5p directly targeted HDAC1 in HUVECs (Fig. 5b). As shown in Fig. 5c, d, anti-miR-135b-5p transfection induced HDAC1 mRNA and protein expression, inversely, overexpression of miR-135b-5p inhibited HDAC1 mRNA and protein expression in HUVECs. Moreover, ox-LDL inhibited HDAC1 mRNA and protein expression in HUVECs (Fig. 5e, f). As shown in Fig. 5g, h, circRSF1 sponged miR-135b-5p to modulate HDAC1 mRNA and protein expression.

### Knockdown of HDAC1 could reverse the effects of anti-miR-135b-5p on cell proliferation, apoptosis and inflammation of ox-LDL-treated HUVECs

**Fig. 6:**
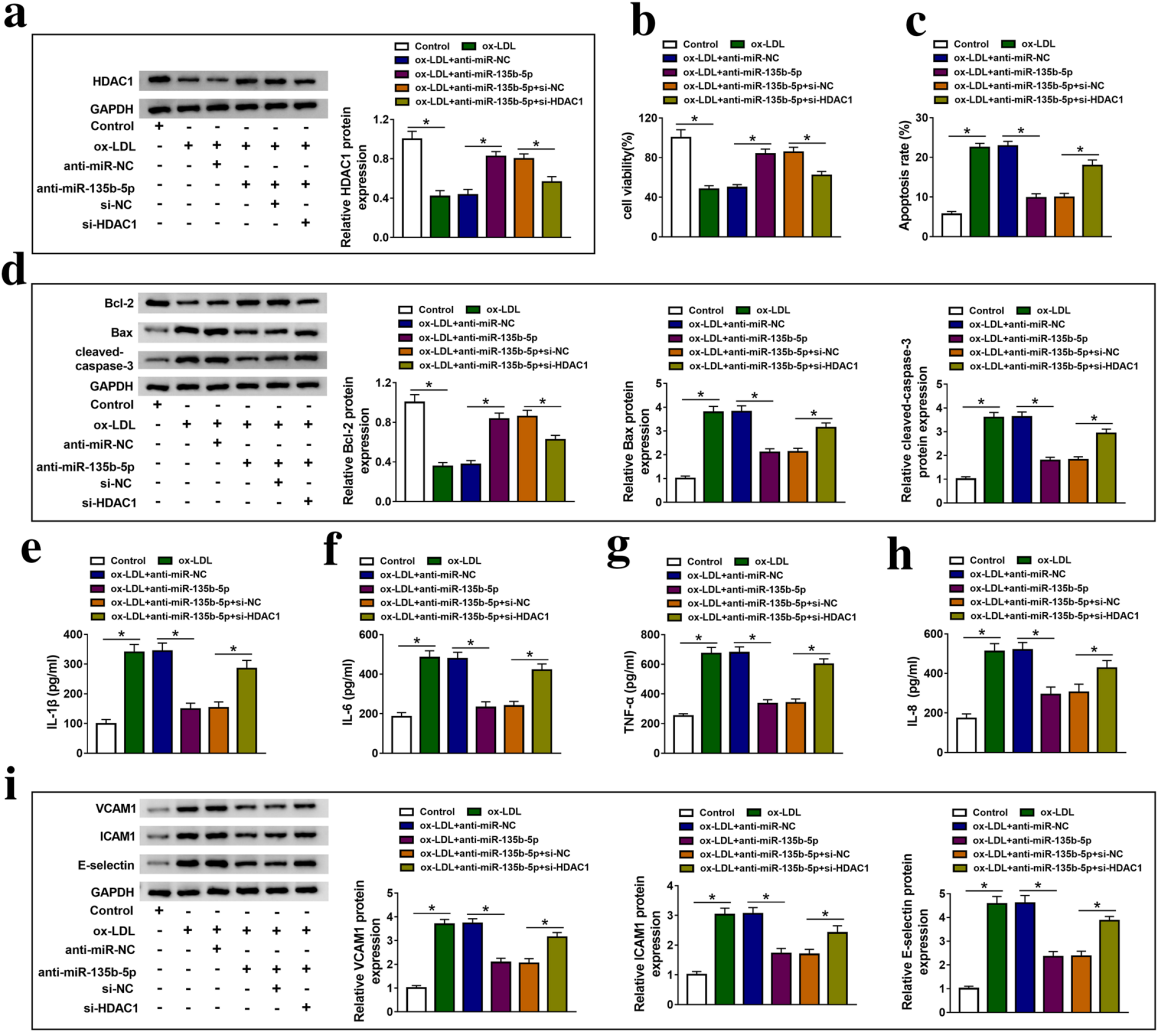
Knockdown of HDAC1 could reverse the effects of anti-miR-135b-5p on cell proliferation, apoptosis and inflammatory of ox-LDL-treated HUVECs. **a** The expression of HDAC1 was measured in control, ox-LDL, ox-LDL + anti-miR-NC, ox-LDL + anti-miR-135b-5p, ox-LDL + anti-miR-135b-5p + si-NC and ox-LDL + anti-miR-135b-5p + si-HDAC1 groups using western blot, the measurements of the following indicators are grouped in this experiment **b** MTT assay was applied to measure cell proliferation. **c** Flow cytometry was used to detect cell apoptosis. **d** Western blot assay detected the protein expression of Bcl-2, Bax and cleaved-caspase-3. **e**–**h** ELISA was used to assess the level of IL-1β, IL-6, TNF-α and IL-8. **i** Western blot assay detected the protein expression of VCAM1, ICAM1 and E-selectin. ‘ + ’ was added, ‘−’ was blank*. Each experiment was performed in triplicate *p* < 0.05

To further investigate the function between miR-135b-5p and HDAC1 in ox-LDL-treated HUVECs, we co-transfected anti-miR-135b-5p with si-NC or si-HDAC1 into ox-LDL-treated HUVECs and the results demonstrated that si-HDAC1 transfection could decrease the protein expression of HDAC1 induced by anti-miR-135b-5p in ox-LDL-treated HUVECs (Fig. 6a). MTT assay and flow cytometry showed that inhibition of miR-135b-5p induced cell proliferation and decreased apoptosis in ox-LDL-treated HUVECs, which was impaired by downregulating HDAC1 expression (Fig. 6b, c). More than that, anti-miR-135b-5p transfection induced Bcl-2 protein expression, and reduced Bax and cleaved-caspase-3 protein expression in ox-LDL-treated HUVECs, which was reversed by si-HDAC1 transfection (Fig. 6d). Furthermore, knockdown of HDAC1 could weaken the suppressive effects of low miR-135b-5p expression on the levels of IL-1β, IL-6, TNF-α and IL-8, and the protein expression of VCAM1, ICAM1 and E-selectin in ox-LDL-treated HUVECs (Fig. 6e–i). Therefore, knockdown of HDAC1 could reverse the effects of anti-miR-135b-5p on cell proliferation, apoptosis and inflammation of ox-LDL-treated HUVECs.

## Discussion

CircRNA plays an important role in the occurrence and progression of diseases, including various cancers, Alzheimer's disease and cardiovascular diseases [33–36]. For example, abnormally expressed circRNAs were found in AS, hepatocellular carcinoma, radioresistant esophageal cancer cells, bladder carcinoma and hypertension, implying that circRNA might be closely associated with disease pathogenesis [37–41]. Moreover, circRNA was involved in the cellular process in diseases, including cell apoptosis, metastasis, invasion, migration, proliferation and inflammation [42–45]. For example, circ_0001564 affected cell proliferation and apoptosis in osteosarcoma [46]. Elevated circ_100876 was found in non-small cell lung cancer and was related to the prognosis [47]. Not only that, in rabbit AS, analysis of circRNA expression patterns and regulatory networks proved that circRNA was an important regulator of the pathogenesis of AS [37]. In this paper, circRSF1 was decreased in AS (ox-LDL-treated HUVECs). Overexpression of circRSF1 promoted the proliferation of AS and inhibited apoptosis and inflammatory response. Therefore, we thought that circRSF1 was a suppressor of the pathogenesis of AS.

MicroRNAs (miRNAs) are usually present as sponges for circRNAs and can be important linkers between circRNAs and the target mRNAs regulation network [48–50]. For example, circ_0010729 regulated cell proliferation and apoptosis through sponging miR-186 via modulating HIF-α in vascular endothelial cell [42]. Interestingly, miR-135b-5p was widely related to cell progression in a variety of diseases and also played a certain role in AS [25–27, 51, 52]. For example, miR-135b-5p was highly expressed in patients with atherosclerosis, and miR-135b-5p promoted endothelial cell proliferation and migration in atherosclerosis [27]. MiR-135b was up-regulated in atherosclerotic mice, and upregulation of miR-135b expression could promote cell apoptosis and inflammatory response, inhibit cell proliferation, and reduce macrophage autophagy [53]. In this paper, we demonstrated that miR-135b-5p was a target miRNA for circRSF1 and was involved in cell proliferation, apoptosis and inflammation in AS.

Furthermore, we also predicted and demonstrated a new target mRNA of miR-135b-5p, HDAC1. Consistent with previous studies, HDAC1was inhibited by ox-LDL treatment in AS [24, 54]. HDAC1 has been extensively documented to be involved in the occurrence and development of a variety of diseases, especially proliferation and apoptosis [30, 31, 55, 56]. In breast cancer, overexpression of HDAC1 affected cell progression through negative regulation of estrogen receptor alpha [57]. In this paper, we also found that after the silencing of HDAC1, the promotion effect of anti-miR-135b-5p on AS could be restored. Therefore, circRSF1/miR-135b-5p/HDAC1 axis has proven to be an important regulatory network in the pathogenesis of AS. However, this mechanism needs to be refined by in vivo experiments.

**Fig. 7:**
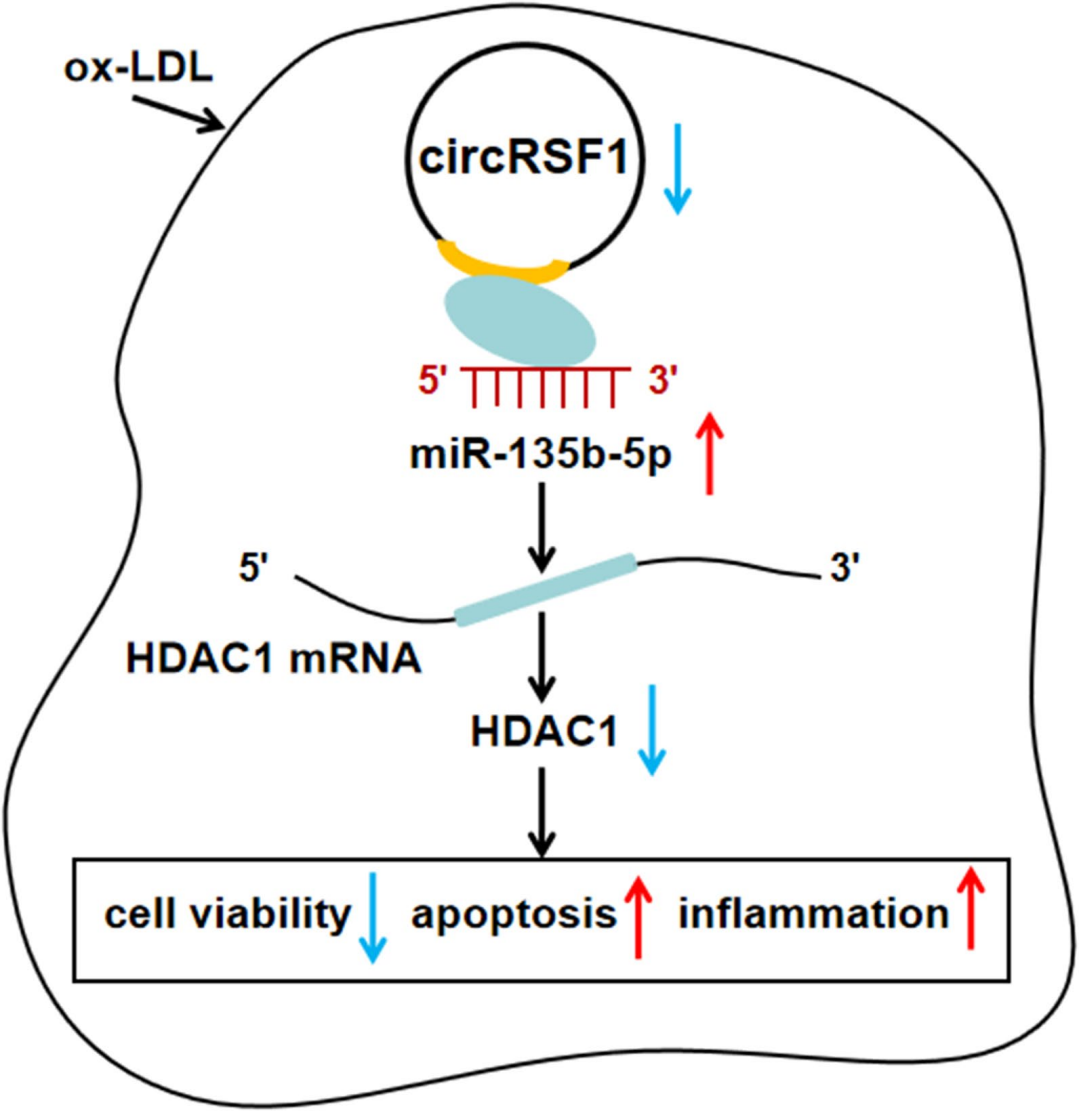
A potentially working model of circRSF1 in cell progression in ox-LDL-treated HUVECs

In summary, circRSF1 had been proved to be a suppressor of the pathogenesis of AS. we proposed a potentially working model that circRSF1 modulated ox-LDL induced vascular endothelial cells proliferation, apoptosis and inflammation through sponging miR-135b-5p via targeting HDAC1 in AS (Fig. 7a), providing a new regulatory network and a novel therapeutic target in AS.

## Conclusion

In summary, circRSF1 was a suppressor of the pathogenesis of AS cells. Furthermore, circRSF1 modulated ox-LDL-induced vascular endothelial cells proliferation, apoptosis and inflammation through sponging miR-135b-5p via targeting HDAC1 in AS, providing a new regulatory network and a novel therapeutic target in AS.

## Data Availability

All data generated or analyzed during this study are included in this article.

## Abbreviations

AS: Atherosclerosis
HDAC1: Histone deacetylase 1
Bax: BCL2-associated X
VCAM1: Vascular cell adhesion molecule 1
ICAM1: Intercellular cell adhesion molecule-1
LDL: Low-density lipoprotein
Ox-LDL: Oxidative (ox)-LDL
circRSF1: Circular RNA remodeling and spacing factor 1
HUVECs: Human umbilical vein endothelial cells
